# A systematic review and meta-analysis of lung cancer risk prediction models

**DOI:** 10.2340/1651-226X.2025.42529

**Published:** 2025-05-12

**Authors:** Ghida Khalife, Matilda Nilsson, Lotta Peltola, Juho Waris, Antti Jekunen, Riikka-Leena Leskelä, Heidi Andersén, Mikko Nuutinen, Eija Heikkilä, Susanna Nurmi-Rantala, Paulus Torkki

**Affiliations:** a Department of Public Health, University of Helsinki, Helsinki, Finland; b Department of Oncology, Vaasa Central Hospital, Vaasa, Finland; c Cancer Clinic, Vaasa Central Hospital, Vaasa, Finland; d Faculty of Medicine, Oncology Department, University of Turku, Turku, Finland; e Nordic Healthcare Group, Helsinki, Finland; f MSD Finland, Espoo, Finland; g Faculty of Medicine and Health Technology, Tampere University, Tampere, Finland

**Keywords:** Lung cancer, risk prediction model, screening, early detection, review

## Abstract

**Background:**

Lung cancer (LC) remains the leading cause of cancer-related mortality worldwide. Early detection through targeted screening significantly improves patient outcomes. However, identifying high-risk individuals remains a critical challenge.

**Purpose:**

This systematic review evaluates externally validated LC risk prediction models to assess their performance and potential applicability in screening strategies.

**Methods:**

Of the 11,805 initial studies, 66 met inclusion criteria and 38 published mainly between 2020 and 2024 were included in the final analysis. Model methodologies, validation approaches, and performance metrics were extracted and compared.

**Results:**

The review identified 18 models utilising conventional machine learning, six employing neural networks, and 14 comparing different predictive frameworks. The Prostate Lung Colorectal and Ovarian Cancer Screening Trial (PLCOm2012) demonstrated superior sensitivity across diverse populations, while newer models, such as Optimized Early Warning model for Lung cancer risk (OWL) and CanPredict, showed promising results. However, differences in population demographics and healthcare systems may limit the generalisability of these models.

**Interpretation:**

While LC risk prediction models have advanced, their applicability to specific healthcare systems, such as Finland’s, requires further adaptation and validation. Future research should focus on optimising these models for local contexts to improve clinical impact and cost-effectiveness in targeted screening programmes.

**Systematic review registration:**

PROSPERO CRD42022321391.

## Introduction

Lung cancer (LC) stands as the deadliest cancer globally, contributing to 18% of all cancer deaths in 2020, surpassing both colorectal and liver cancer. With over two million new LC cases yearly, it ranks among the most prevalent cancers worldwide [1]. In Finland, 5-year survival rates for 2016–2020 were 16.4% for men and 25.5% for women, lagging behind other Nordic countries where men’s survival ranged from 23.8% to 26.6% and women’s from 30.6% to 33.2% [2]. Given its lethality, early detection represents the most effective strategy for improving survival rates, which could be achieved through the screening of high-risk individuals [3].

Earlier reviews have highlighted various risk prediction models designed to identify high-risk individuals, encouraging them to undergo further diagnostic investigation. Field et al. [4] conducted one of the earliest literature reviews in 2013, featuring five main models: the Bach, Spitz, Liverpool Lung Project (LLP), Prostate Lung Colorectal and Ovarian Cancer Screening Trial, and European Prospective Investigation into Cancer and Nutrition (EPIC) models. The PLCOm2012 model exhibited superior discrimination with an area under the curve (AUC) of 0.84 across all subjects. Subsequent reviews included Gray et al. [5], who identified 31 articles and 25 distinct models, with the PLCOm2012 and Hoggart models demonstrating the best performance. Schmidt-Hansen et al. [6] conducted a systematic review based on the United Kingdom (UK) primary care data, including seven non-externally validated studies. They concluded that there is insufficient evidence to recommend any model. In 2019, Tang et al. [7] reviewed 22 studies, reporting high overall prediction accuracy with AUCs ranging from 0.57 to 0.87. A more recent review by Wu et al. [8] compared pulmonary nodules’ risk prediction models, including 43 models, most of which lacked external validation but generally demonstrated good discrimination.

The systematic review by Toumazis et al. in 2020 [9] holds particular relevance to our investigation, as it focuses on the application of LC risk prediction models and provides details on their predictive performances. Within their comprehensive analysis of 47 articles, they identified 15 models incorporating biomarkers and 28 assessing the malignancy of pulmonary nodules. These models draw on various factors, mainly age, sex, and smoking history. Besides, 27 models include sociodemographic and socioeconomic factors. The primary objective of most models is to identify individuals who could benefit from screening. The review accumulates important evidence indicating that a risk prediction model may surpass selection criteria based solely on age and smoking history. However, the development of advanced methodologies for individual risk assessment has been rapid in recent years, and there is a need for evaluation of the performance of the most recent models.

In this review, our primary focus is to identify externally validated risk prediction models not previously mentioned in Toumazis et al.’s review. We aim to bring into focus models that could enhance early LC diagnosis and facilitate the selection of high-risk individuals for a targeted LC screening programme. The goal is to identify the most effective prediction model that could be suitable for implementation in real-world environment to enable bigger share of curative treatment.

## Methods

### Literature review

We conducted a search in the MEDLINE database via PubMed Library for studies in English published before March 2024. The protocol for this systematic review was registered on PROSPERO (CRD42022321391). We used combinations of the following terms: ‘lung cancer’, ‘lung neoplasm’, ‘risk prediction’, ‘risk assessment’, ‘model’ and ‘tool’. We required the terms ‘lung cancer’ or ‘lung neoplasm’ to appear in either the title or abstract.

Four reviewers (L.P., M.N., G.K., and P.T.) independently reviewed the articles based on the title and abstract. All types of studies that develop, update, or validate a risk prediction model or compare existing prediction models for LC will be considered. If there was an updated version of a pre-existing model, we included the latest version. Articles were excluded if they met at least one of the following criteria: (1) the study was not related to LC; (2) the model didn’t assess risk on an individual level; (3) the model had not been externally validated; (4) the full article could not be obtained; (5) the article was not written in English language; (6) the article wasn’t published in a peer-reviewed journal; and (7) if the article is a review, letter, editorial, or commentary.

Once the studies had been initially included or excluded based on the title and abstract, four reviewers (L.P., M.N., G.K., and P.T.) screened the remaining articles based on the full text, using the same inclusion and exclusion criteria. Differences in opinion were resolved through discussion and consensus.

### Data extraction

Two reviewers (G.K. and M.N.) conducted data extraction for eligible articles independently. This process involved the collection of various study characteristics, including study design, setting, population, model type, risk factors, risk prediction type, and the time period for risk prediction. Additionally, we reported on predictive performance, including model validation and performance on independent cohorts, sensitivity, and specificity.

## Results

**Figure 1:**
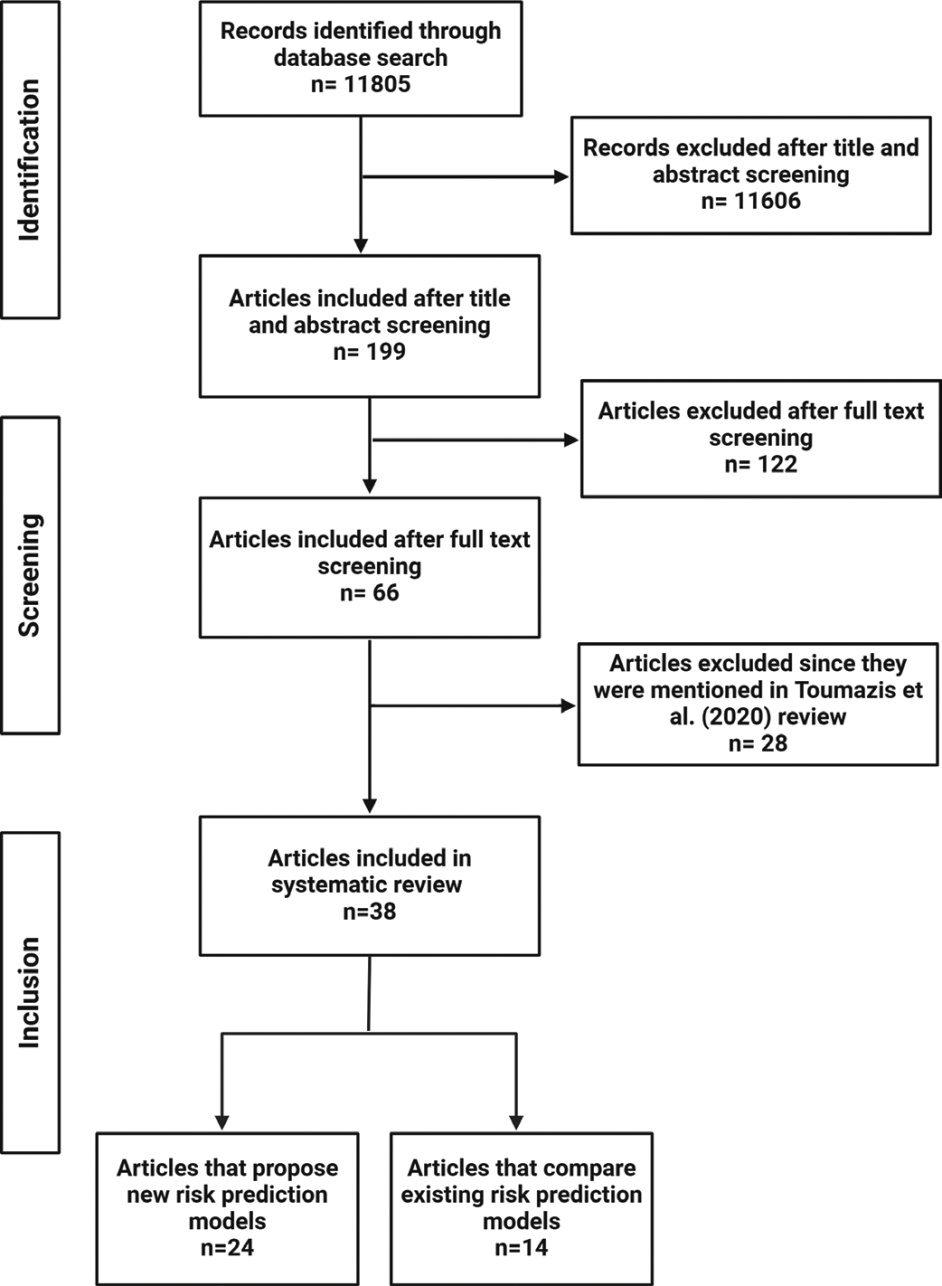
Selection process of records applied in this systematic review.

The initial literature search yielded 11,805 scientific articles. After reviewing the title and abstract of each article, we excluded those that did not propose, validate, or compare LC risk prediction models, reducing the number of articles to 199. We conducted a full-text review and included 66 manuscripts that met the inclusion criteria (Figure 1).

To ensure our review included novel contributions not covered in similar reviews, such as that by Toumazis et al., we excluded 28 articles and included 38 unique studies. We categorised these articles into two groups: (1) those that proposed new risk prediction models (*n* = 24), and (2) those that focused on the comparison of existing risk prediction models (*n* = 14). Table 1 provides a summary of the articles that propose a new model, detailing the model type, prediction type, and whether external validation using real-world data was performed.

**Table 1:** Summary of included articles that propose a new model.

Model type	Total	Prediction type	Number of articles	External validation (Real-World Data)
Conventional ML methods	18	LC risk incidence ≤ 6 years	13	10
		LC incidence > 6 years	6	
Neural network methods	6	LC risk incidence ≤ 6 years	3	
		LC incidence > 6 years	3	

LC: lung cancer; ML: Machine Learning.

### Risk prediction models suitable for selecting high-risk individuals for screening

We identified a total of 24 articles in this category. Of these, 18 employ conventional methods, such as logistic regression models, while six utilise neural network models. All models have undergone external validation using independent datasets. Table 2 includes models that employ conventional methods while Table 3 summarises the models that use neural network methods for developing a risk prediction model.

**Table 2:** Lung cancer risk prediction models intended to select high-risk individuals for screening, utilising conventional machine learning methods.

Ref. No.	Author (year)	Risk prediction model	Predictors	Study design	Model type	Targeted population	Sample size	Prediction type	External validation	Sensitivity	Specificity
[18]	Hung et al. (2021)	PLCO_m2014_+PRS	Age, race, education level, Body Mass Index (BMI), Chronic obstructive pulmonary disease (COPD), history of cancer, family history of LC, smoking (status, intensity, duration, quit time)	Cohort	Cox proportional hazards regression	General population	23,127	The 5-year cumulative and absolute risk of developing LC	AUC: 0.832 AUC in never-smokers: 0.687		
[25]	Park et al. (2021)	Korean LC risk model (NHIS data)	Age, sex, smoking (pack-years, status, cessation), physical activity, alcohol consumption, BMI, COPD, emphysema, pneumoconiosis, interstitial pulmonary disease	Cohort	Cox proportional hazards model	Ever-smokers	969,351	6.6-year LC incidence risk	AUC: 0.816	60.1%	83.0%
[26]	Chien et al. (2020)	Taiwanese Never-Smoking Females Lung Cancer Risk Models-Simplified Questionnaire (TNSF-SQ)	Age, education, BMI, family history of LC, COPD	Age-matched case–control	Multivariate logistic regression model	Non-smoking females	8,283	6-year LC incidence risk	AUC: 0.714(95% CI, 0.660–0.768)		
[19]	van ‘T Klooster et al. (2020)	Cardiovascular disease (CVD) model	Age, sex, smoking, weight, height, alcohol use, anti-platelet use, diabetes, C-reactive protein	Cohort	Regression model	CVD patients	16,602	4-year, 10-year, and total LC incidence risk	C-statistic: 0.74 (95% CI, 0.70–0.77)		
[46]	Usher-Smith et al. (2019)	Lifestyle-based model	Age, smoking status, BMI, physical activity, fruit and vegetable intake	Cohort	Cox proportional hazard regression	General population	23,768	10-year absolute LC incidence risk	AUC: 0.80 for men (95% CI, 0.77–0.83) and 0.82 for women (95% CI, 0.76–0.87)		
[27]	Charvat et al. (2018)	Japan Public Health Center-based study (JPHC)	Age, gender, smoking (pack-years, age at initiation, and time since cessation)	Cohort	Cox proportional hazard regression	General population	106,662	10-year cumulative risk	C-index: 0.772		
[16]	Cheng et al. (2017)	Genetic risk predictor model	38 different genetic variants, genetic risk score (GRS), smoking status	Two-stage case control study	LASSO penalized regression	Asian and European populations	9,329		AUC: 0.558 (95% CI, 0.540–0.576)		
[17]	Titulaer et al. (2011)	DELTA-P score for Lambert-Eaton myasthenic syndrome (LEMS)	Age at onset, smoking behaviour, weight loss, bulbar involvement, erectile dysfunction, Karnofsky performance status	Cohort	Multivariate logistic regression	LEMS patients	107	Short-term prediction of small-cell LC	AUC-ROC: 0.946		
[10]	Maddison et al. (2020)	DELTA-P score validation	Age at onset, smoking behaviour, weight loss, bulbar involvement, erectile dysfunction, Karnofsky performance status	Cohort	Multivariable logistic regression	LEMS patients	87	Short-term prediction of small-cell LC	AUC-ROC: 0.825 (95% CI, 0.739–0.91)		
[41]	Callender et al. (2023)	Lung cancer incidence (UCL-I) and death (UCL-D)	Age, smoking duration, pack-years	Cohort	Logistic regression, random forests, and Gradient Boosting	Ever-smokers and high-risk ever-smokers	243,330	5-year incidence risk	AUC: 0.787 (95% CI, 0.771- 0.802)	83.9%	
[28]	Jacobsen et al. (2023)	Risk Chart	Age, sex, education, chronic obstructive pulmonary disease, family history of LC, smoking status and cumulative smoking, second-hand smoking, occupational exposures to dust and fume, body mass index, lung function, plasma C-reactive protein, and AHRR (cg05575921) methylation	Cohort	Logistic regression and Poisson regression	Former or current smokers	6,820	10-year incidence risk	AUC-ROC: 0.70 (95% CI: 0.69–0.72)	74%	70%
[31]	Feng et al. (2023)	4- marker protein-based model	4 specific markers: CEACAM5, MMP12, IL6, and CDCP1. CEACAM5	Case-control study	LASSO logistic regression	General population with a history of smoking	1,718	3-year incidence risk	AUC: 0.75 (95% CI= 0.70- 0.81)		
[12]	Ma et al. (2023)	Lung cancer risk score (LCRS)	Age, residential location, education, BMI, smoking status, smoking intensity, physical activity, emphysema, bronchitis, frequent cough, family history of cancer	Cohort	Cox regression model	Ever-smokers and never-smokers in China	496,241	3-, 5-, 6-, and 10-year incidence risk	AUC-ROC: For ever-smokers: 0.774 For never-smokers: 0.759	73.08%	66.33%
[30]	Chandran et al. (2023)	Optum Electronic Health Record (EHR)	Age, smoking, race, ethnicity, and diagnosis of chronic obstructive pulmonary disease	Cohort	LASSO regression model	General population	4.7 million	3-year incidence risk	AUC: MERCY EHR: 0.81 Optum Claims: 0.72		
[20]	Pan et al. (2023)	Optimized Early Warning model for Lung cancer risk (OWL)	Age, race, smoking (status, duration, pack-years), education level, family history of cancer, diabetes, chronic bronchitis, emphysema, COPD	Cohort	eXtreme Gradient Boosting (XGBoost)	General population and ever-smokers	323,344	5-, and 6- year incidence risk	Validation set 1: AUC _5-year_ = 0.838 AUC _6-year_ = 0.842		
[11]	Liao et al. (2023)	The CanPredict model	Sociodemographic characteristics (age, sex, ethnicity, Townsend score), lifestyle factors (BMI, smoking and alcohol status), comorbidities, family history of LC, and personal history of other cancers.	Cohort	Cox proportional-hazards model	Ever-smokers and never-smokers	12.99 million	5-, and 6- year incidence risk	C-statistic: Women: 0.869 Men: 0.878		
[13]	Wang et al. (2023)	China NCC-LC_m2021_	Cigarette smoking, occupational exposure to hazardous substances, frequent exercise, chronic respiratory diseases, a family history of LC, dietary intake of fresh vegetables in the last year, and passive smoking	Cohort	Multivariable Cox Regression model	Ever-smokers and never-smokers	554,578	3-year incidence risk	AUC-ROC in Validation A: Ever-smokers: 0.698 Never-smokers: 0.728	68%	66%
[29]	Jantzen et al. (2023)	PLCOm2012 in CARTaGENE cohort	Age, gender, education, BMI, COPD history, cancer history, family history of LC, smoking (status, duration, quit time)	Cohort	Logistic regression model	Ever-smokers	11,652	6-year incidence risk	C-statistic: 0.727 (95% CI 0.679–0.770)	52.3%	81.6%

AUC: area under the curve; AUROC: Area Under the Receiver Operating Characteristic Curve; CI: confidence interval; LC: lung cancer; NHIS: The Korean National Health Insurance Service; PRS: Polygenic risk score.

**Table 3:** Lung cancer risk prediction models intended to select high-risk individuals for screening, utilising neural network methods.

Ref. No.	Author	Risk prediction model	Predictors	Study design	Model type	Targeted population	Sample size	Prediction type	External validation	Sensitivity	Specificity
[14]	Lee et al. (2021)	Deep Neural Network (DNN) model	13 diseases included lung-, diabetes-, hypertension-, and heart-related diseases	Cohort	DNN	Taiwanese population	2,000,000	10-year incidence	AUROC: 0.874 (95% CI, 0.8604–0.8885)	72.4%	85%
[21]	Yeh et al. (2021)	Convolutional Neural Network (CNN) model	Age, sex, medication, diagnoses	Cohort	CNN	Taiwanese population	1,434,774	1-year incidence risk	AUC: 0.898	80.5%	82.5%
[15]	Lu et al. (2020)	CXR-LC	Chest x-ray image, age, sex, current smoker	Cohort	CNN	Never, former, and current smokers	53,414	12-year incidence risk	AUC: 0.755	74.9%	63.6%
[22]	Raghu et al. (2022)	CXR-LC+ Electronic Medical Record (EMR)	Age, sex, current cigarette smoking status, and a chest radiograph image	Cohort	Deep learning CNN	Current and former smokers	14,737	6-year incidence risk			
[23]	Chen et al. (2023)	ECRs	Age, gender, history of any cancer, related screens, related symptoms	Cohort	Transformer	General population	82,092	3-year incidence risk	AUC: 0.738		
[24]	Lee et al. (2022)	DL+ USPSTF2021	Age, sex, smoking status (current or former smoker) with pack-year information	Cohort	CNN	Current and former smokers	19,488	Up to 12-year incidence risk	AUC: 0.68	90%	27%

AUROC: Area Under the Receiver Operating Characteristic Curve; CXR-LC: Deep learning tool using Chest Radiographs; ECRs: Electronic Claims Records; DL: Deep learning.

The sample sizes used to train the models in the reviewed studies varied significantly, ranging from 87 participants [10] to cohorts as large as 12.99 million [11], utilising data from extensive electronic databases. While the majority of the articles target ever-smokers, five out of the 24 studies included never-smokers in their model development [11–15]. Besides, age, smoking history, body mass index (BMI), chronic obstructive pulmonary disease (COPD) status, education level, family history of LC, and gender emerged as the most common predictors across these models.

External validation of the models showed a diverse range of AUC values, spanning from 0.558 in the study by Cheng et al. [16] to 0.946 in the model developed by Titulaer et al. [17]. Most models achieved AUCs around 0.7. Cheng et al. employed a case-control study to develop a risk score integrating genetic variants into an existing prediction model, yielding an AUC increase from 0.610 to 0.690 in their testing set. However, the genetic model alone exhibited a less robust performance with an AUC of 0.558 in the validation cohort. Another important study by Hung et al. [18] focused on the impact of genetics on LC risk stratification. They introduced a polygenic risk score (PRS) into the existing PLCOm2014 model to assess potential improvements in performance. Their findings revealed that the addition of the PRS did not alter the overall AUC, which was 0.832 with the PRS and 0.828 without it.

For the prediction of small-cell LC, Titulaer et al. [17] developed the DELTA-P score for predicting small-cell LC in Lambert-Eaton Myasthenic Syndrome (LEMS) patients, achieving a high AUC of 0.946 in their validation study. This model was later validated by Maddison et al. in 2020 [10], confirming its clinical validity with an AUC of 0.825 based on clinical features of 87 newly diagnosed LEMS patients. Another high-risk group includes patients with cardiovascular disease, for whom van’t Klooster et al. [19] developed a risk prediction model for 4-year, 10-year, and total cancer risk. During the validation phase, the LC model exhibited an AUC of 0.74.

Other models that achieved high AUCs include the CanPredict model [11] and the Optimized Early Warning model for Lung cancer risk (OWL) [20]. When it comes to neural networks, Yeh et al. [21] developed a Convolutional Neural Network (CNN) model that predicts LC risk within 1 year using data extracted from electronic medical records (EMRs). This approach achieved strong performance metrics in the test set, including an AUC of 0.898, sensitivity of 80.5%, and specificity of 82.5%. The CNN models have gained popularity in recent years [15, 21–24], with some having the ability to predict LC incidence up to 12 years in advance.

Some machine learning models incorporate questionnaires to gather additional data not typically available in routine clinical records, such as smoking history, family medical history, occupational exposures, and lifestyle factors like physical activity and alcohol consumption [13, 20, 25–29], while other models leverage real-world data from health records [11, 15, 21, 23, 30].

In addition, several studies developed prediction models tailored to specific populations [12, 13, 25–27]. Charvat et al. [27] created a model specifically for the Japanese population, which demonstrated strong discrimination with a c-index of 0.772 in the external validation cohort. Chien et al. [26] developed the Taiwanese Never-Smoking Females Lung Cancer Risk Models-Simplified Questionnaire (TNSF-SQ) model specifically for non-smoking females in Asia. Park et al. [25] designed a prediction model for the Korean population, assessing its performance against existing models and achieving an AUC of 0.816 in the validation cohort. For the Chinese population, Wang et al. [13] developed and validated the China National Cancer models (NCC-LCm2021), which predict 3-year LC incidence regardless of smoking status. The AUCs in the two validation cohorts were 0.698 and 0.673 for never-smokers, and 0.728 and 0.752 for ever-smokers. Additionally, Ma et al. [12] constructed a LC risk score (LCRS) that included both ever-smokers and never-smokers, with AUCs of 0.774 and 0.759, respectively.

While numerous models have been designed to detect LC using biomarkers, this review highlights two specific articles focused on biomarkers predicting LC incidence. Feng et al. [31] developed a proteomics-based risk model, demonstrating that circulating proteins are potential predictors of LC incidence and outperform standard risk prediction models. Similarly, Jacobsen et al. [28] created and validated a straightforward risk chart incorporating Aryl-hydrocarbon Receptor Repressor (AHRR) methylation as a predictor for estimating 10-year LC risk. Their findings indicate that including AHRR methylation in screening eligibility criteria effectively identifies smokers who would benefit most from screening.

### Risk prediction models comparing existing models

We identified 14 additional articles that compare the performance of various LC risk prediction models. Table 4 summarises these models and highlights the best-performing ones. Remarkably, seven out of the 14 articles reported that PLCOm2012 was the best model.

**Table 4:** Articles comparing existing risk prediction models.

Ref. No.	Author	Cohort (*n*)	Number of models compared	Best model	Predictive performance of best model
[32]	Jacobsen et al. (2022)	Copenhagen Heart Study (*n* = 9,206)	7	PLCOm2012	PPV: 3.2%
[33]	Robbins et al. (2021)	3 different cohorts (*n* = 273,789)	6	LCDRAT	AUC: 0.82
[34]	Bhardwaj et al. (2022)	ESTHER cohort in Germany (*n* = 4812 ever-smokers)	11	Bach	AUC: 0.766
[35]	Ostrowski et al. (2021)	MOLTEST BIS (*n* = 6,631)	3	PLCOm2012	AUC: 0.717
[40]	Field et al. (2021)	Questionnaire (*n* = 75,958)	2	LLPv3	AUC: 0.81
[47]	O’Dowd et al. (2022)	Clinical Practice Research Datalink (*n* = 842,109)	2	PLCOm2012	AUC: 0.679
[36]	Pasquinelli et al. (2021)	Chicago Race Eligibility for Screening Cohort (*n* = 883)	2	PLCOm2012	Sensitivity: 79.1%
[48]	Kats et al. (2021)	Electronic Health Records (*n* = 37,422)	2	LCRAT	AUC: 0.72
[42]	Lebrett et al. (2020)	Ever-smokers aged 54–77 in Manchester	2	N/A	N/A
[37]	Pu et al. (2022)	INHALE epidemiology study (*n* = 2369)	4	PLCOm2012	Sensitivity: 68%
[38]	Walter et al. (2023)	DZL data warehouse in Germany (*n* = 3623)	5	PLCOm2012	Sensitivity: 72.4%
[49]	Smith et al. (2022)	*N* = 120 from a Canadian province	3	LLPv2	Sensitivity: 80.8%
[39]	Williams et al. (2022)	*N* = 447	2	PLCOm2012	Sensitivity: 71.4%
[50]	Nguyen et al. (2024)	*N* = 44,831 ever-smokers from 10 Norwegian cohorts	3	HUNT LCM	Sensitivity: 70.7%

PPV: Positive Predictive Value; AUC: area under the curve; HUNT LCM: Helseundersøkelsen i Nord-Trøndelag Lung Cancer Model.

Furthermore, three of the 14 articles compared the performances of more than six LC risk prediction models [32–34]. Jacobsen et al. [32] aimed to enhance LC screening eligibility criteria by externally validating seven criteria (DANTE, DLCST, ITALUNG, LUSI, NELSON, PLCOM2012) and introducing AHRR (cg05575921) methylation. Some models exhibit better calibration but poorer discrimination, while others maintain high sensitivity or specificity. Similar to the more recent study by Jacobsen et al. [28], the results demonstrated that the inclusion of AHRR (cg05575921) methylation significantly reduced the screening burden by 21.9% and increased the specificity to 84.0%.

Robbins et al. [33] evaluated the calibration and discrimination of six different risk models (PLCOm2012, LLPv2, LLPv3, Lung Cancer Death Risk Assessment Tool (LCDRAT), Lung Cancer Risk Assessment Tool (LCRAT), Bach), and assessed their ability to accurately predict LC risk to assess their ability to accurately predict LC risk. They found that all six models over-predicted risk in groups with high socioeconomic status. The LLPv2 model had the worst calibration, while the LLPv3 model had the best calibration but poor discrimination. In a comprehensive study, Bhardwaj et al. [34] evaluated 11 models over a 17-year follow-up, finding LCRAT, LCDRAT, and Bach models performed best in predicting LC incidence and mortality, suggesting these models could enhance screening programmes.

Among the 14 articles comparing different LC risk prediction models, seven identified PLCOm2012 as the superior model. Ostrowski et al. [35] demonstrated that the PLCOm2012 model effectively reduces the proportion of eligible patients for inclusion in a screening programme while maintaining a high detection rate of LC cases. Pasquinelli et al. [36] found that PLCOm2012 outperformed USPSTF2020 criteria in selecting a racially diverse population for screening. Similarly, Pu et al. [37] found that PLCOm2012 had a sensitivity of 68%, compared to 65% for USPSTF2021 and 49% for USPSTF2013. Thus, while racial disparities were evident with USPSTF2013, they were absent with PLCOm2012 and the other models. Walter et al. [38] evaluated the effectiveness of various selection criteria in identifying LC patients for screening in a German cohort and found that the adapted PLCOm2012 model selected the highest proportion of LC patients. Lastly, Williams et al. [39] compared the sensitivity of USPSTF2013 and PLCOm2012 in detecting LC cases eligible for screening, finding that PLCOm2012 was more sensitive, particularly among Black individuals and women.

Field et al. [40] assessed the discriminatory and predictive power of the existing LLPv2 model, and proposed a new validation model, LLPv3, which may be more suitable for a nationwide population. LLPv3 was found to effectively identify high-risk individuals compared to LLPv2, maintaining similar discriminatory power without overestimating like LLPv2.

## Discussion

### Overview of models

This systematic review aimed to evaluate existing LC risk prediction models to screen high-risk individuals. The analysis included a comprehensive review of studies published up to March 2024, resulting in the identification of 38 relevant studies for inclusion in this review, with 18 utilising conventional machine learning methods, six employing neural network models, and 14 comparing different risk prediction models. The primary focus was on assessing the predictive performance of these models in an independent cohort. There was considerable variability between the identified lung models in terms of methodology, risk factors considered, and predictive accuracy. Several models demonstrated promising discriminative ability in distinguishing high-risk individuals, such as CNN risk prediction model. Among the compared models, PLCOm2012 emerged as the most consistently effective across multiple studies. While the systematic review by Toumazis et al. [9] provides a broad evaluation of LC risk prediction models, our systematic review builds upon their work by focusing exclusively on externally validated models, assessing their real-world feasibility and clinical utility, and identifying the most suitable model for implementation.

The models varied in their prediction timelines, ranging from diagnosis within 1 year to 12 years. Most studies aimed for a 5 to 6-year prediction horizon. There is an evident increase in models incorporating genetic factors, along with a rise in the number of prediction factors. Newer models often build upon or extend the foundations laid by earlier models, such as the PLCOm2012 or LLP model, while there has been a clear shift from traditional logistic regression models to more advanced technologies like neural networks.

Recent deep learning models offer key advancements over traditional models by using large-scale medical data and identifying complex patterns with minimal manual input [14]. For example, the CNN model analyses more intricate features from EMRs, such as comorbidities and treatment history, improving risk stratification for individuals with multiple pre-existing conditions [21]. Some neural network models, like Deep Learning tool using Chest Radiographs CXR-LC, identify high-risk smokers using imaging data alone, making them more adaptable for settings with incomplete patient histories [15]. Additionally, Deep Neural Networks (DNNs) enhance predictive accuracy by uncovering complex interactions between clinical and demographic variables that conventional models often overlook [15, 22, 24]. These models also integrate seamlessly into EMRs and generate real-time alerts, potentially prompting earlier discussions on LC screening in clinical settings [22]. Together, these innovations make modern deep learning models more precise, scalable, and accessible for diverse healthcare environments.

Although many of the reviewed LC risk prediction models demonstrate strong performance metrics, their applicability to Finnish primary healthcare settings remains uncertain. Most of the models were developed and validated using datasets from populations with different demographic and clinical characteristics, primarily from countries such as China, Korea, the United States of America (US), the UK, which may limit their generalisability to the Finnish context.

### Analysis of various models

#### Complexity and clinical utility

The complexity of a model directly affects its ease of implementation and practicality in real-world settings. For instance, China NCC-LCm2021 stands out for its detailed requirements, involving numerous patient-specific factors [13], which presents challenges for clinical use, particularly in resource-limited settings. The need for extensive data makes it less adaptable for healthcare systems lacking standardised patient information.

In contrast, as UCL-I relies on only three predictors [41], this model is simpler to implement, making it both accurate and user-friendly within clinical workflows. Another model that was developed using readily accessible predictors is the LCRS [12]. This facilitates its straightforward application in clinical practice. The inclusion of an online tool, Lung Cancer Risk Evaluation by Yourself (LCKEY), enables easy calculation of risks and provides personalised lifestyle change suggestions and screening recommendations.

However, simplicity can sometimes come at the cost of missing critical details. While models like CanPredict and PLCOm2012 include numerous variables to enhance predictive accuracy, they simultaneously increase the complexity of data collection and processing.

That said, models like OWL bridge this gap by offering an easy-to-use platform based on well-established questionnaire-based predictors [20], which facilitates and eases their implementation in clinical practice. Moreover, the additional data points used in questionnaires can significantly enhance the model’s ability to predict risk by providing a more comprehensive picture of an individual’s health profile. Lastly, some models, such as the 4-marker protein-based model and the genetic risk prediction model, require complex information [16, 31]. Yet, the relevance of such models that include clinical and biomarker data to the general population is still uncertain.

#### Sensitivity and specificity

The sensitivity and specificity of risk prediction models are crucial in determining their effectiveness in LC screening. For instance, PLCOm2012 demonstrates high sensitivity, excelling in predicting LC risk with minimal loss of detected cases. Its broad applicability across diverse populations further strengthens its role in identifying high-risk individuals [35], and it has been noted for reducing racial disparities in screening [36, 37, 39]. However, despite these strengths, the model tends to overestimate risks in higher-risk groups and underestimate them in lower-risk bands, particularly among individuals with lower socioeconomic status [11, 42].

The OWL model, with its use of the XGBoost algorithm, has demonstrated superior predictive accuracy and calibration compared to the PLCOm2012, PLCOm2014, and LLPv3 across multiple validation sets [20]. Similarly, the CanPredict model [11] achieves excellent discrimination and calibration across different sexes and ethnicities. However, its reliance on routinely collected Electronic Health Records (EHRs) can lead to inaccuracies, as it may miss relevant information more easily gathered in dedicated screening settings. Moreover, the CanPredict model’s ability to predict risk over a 10-year horizon raises concerns about unnecessary screenings, overdiagnosis, and potential patient anxiety, all of which could impact its cost-effectiveness.

The discussed CNN model [21], uses complex features from EMRs and yields a high positive predictive value among older patients with pre-existing lung disease. Nonetheless, the model faces issues with calibration, a common challenge for modern neural networks due to their architectural complexity. Meanwhile, the DNN [14] shows the model’s capability to aid in early diagnosis. Its use of advanced deep learning techniques uncovers predictive features that were previously unrecognised by traditional clinical knowledge.

#### Generalisability

The ability of these models to generalise across different populations is a crucial factor for broad adoption. The PLCOm2012 model shows high generalisability, as it performs well across diverse racial and ethnic groups. However, several models, such as Taiwanese Never-Smoking Females Lung Cancer Risk Models-Simplified Questionnaire (TNSF-SQ), Japan Public Health Center–based study (JPHC), and Korean LC risk model, are tailored specifically for certain populations, limiting their generalisability to other settings. Similarly, LCRAT and LCDRAT show variability in their performance across populations and genders, where the models tend to underperform in selecting women for screening [38]. This issue of limited external validation is also present in the Lung Cancer Incidence (UCL-I ) model, which has been validated only in the UK and the US cohorts [41].

Also, despite its strengths, the Taiwanese DNN model may be geographically constrained due to its data being sourced exclusively from Taiwan [14]. A similar limitation affects the CNN model, which relies on Taiwan National Health Insurance Research Database (NHIRD) data and lacks critical factors like smoking status, tissue histology, and cancer staging, restricts its ability to generalise effectively [21].

In Finland, specific population factors such as high radon exposure [43] and the association between higher education and decreased LC risk [44] could affect the performance of these models. Incorporating these elements into existing models may improve their accuracy for use within the Finnish population. However, given Finland’s lack of established national LC and screening programmes [45], models like UCL-I or OWL, which require fewer predictors or are easier to implement, might be more practical in primary care settings during future screening programme development.

### Assessing the best prediction model

Based on the criteria of accuracy, ease of implementation, and generalisability across different populations, the OWL model emerges as the leading candidate. Its combination of high predictive power, robust discrimination, and straightforward implementation makes it a strong tool for early LC detection. Although further validation in diverse populations is necessary, its current performance exceeds that of other models.

On the other hand, if the focus is on ensuring robustness across diverse populations while incorporating a wide array of predictors, the CanPredict model is a strong alternative. However, it involves some disadvantages such as potential anxiety from long-term predictions and reliance on routine EHRs. Screening programmes are unlikely to depend on current EHRs due to the well-known issues of missing and inaccurately coded predictors.

### Strengths and limitations

The strengths of this systematic review lie in its rigorous inclusion criteria, where only externally validated articles were considered. Besides, the search algorithm incorporated diverse terms, revealing new models not previously mentioned in systematic reviews. While this study provides valuable insights, it is not without limitations. One constraint is the exclusion of prediction models involving CT scans, as well as articles which have information on primary nodules. Besides, the potential for publication bias should be recognised.

### Implications for research and practice

Our findings have significant implications for the development and implementation of LC screening programmes for high-risk individuals. They will be valuable for researchers aiming to create new prediction models, as well as for clinicians and policy makers who are looking into developing future screening strategies. Current models seem to have limited integration of biomarkers, such as proteomics, methylation, and genetic factors. Incorporating these biomarkers could enhance the precision of risk predictions by identifying molecular factors that are not captured by clinical data alone. Future research could focus on exploring such integrations to improve the model’s diagnostic power and ability to identify individuals at the highest risk.

Also, the performance of these models in specific healthcare settings requires further evaluation through local data validation. Adjustments to account for population-specific risk factors, such as those unique to Nordic countries or other regional contexts, are essential to ensure the models’ relevance and effectiveness. Moreover, the availability of relevant data may vary in different countries. Future research should explore the integration of machine learning with biomarker data to tailor models for diverse populations, a crucial step in advancing the next generation of LC screening programmes.

## Data Availability

Data used in this study were derived from publicly available sources and published literature.
